# Combined Disruption of the Thoracic Spine and Costal Arch Fracture: An Indicator of a Severe Chest Trauma

**DOI:** 10.3390/diagnostics12092206

**Published:** 2022-09-12

**Authors:** Stefan Schulz-Drost, Stephan Kloesel, Jan Carsten Kühling, Axel Ekkernkamp, Mustafa Sinan Bakir

**Affiliations:** 1 Department of Trauma Surgery, Helios Hospital Schwerin, Wismarsche Str. 393-397, 19049 Schwerin, Germany; 2 Department of Trauma and Orthopedic Surgery, University Hospital Erlangen, Krankenhausstr. 12, 91054 Erlangen, Germany; 3 Department of Trauma Surgery and Orthopedics, BG Hospital Unfallkrankenhaus Berlin gGmbH, Warener Straße 7, 12683 Berlin, Germany; 4 Department of Trauma and Reconstructive Surgery and Rehabilitative Medicine, Medical University Greifswald, Ferdinand-Sauerbruch-Straße, 17471 Greifswald, Germany; 5 Department of Paediatric Surgery, Medical University Greifswald, Ferdinand-Sauerbruch-Straße, 17471 Greifswald, Germany

**Keywords:** thoracic spine fracture, costal arch fracture, severely injured, indicator injury, intercostal hernia

## Abstract

Blunt high-energy chest trauma is often associated with thoracic and abdominal organ injuries. Literature for a hyperextension-distraction mechanism resulting in a costal arch fracture combined with a thoracic spine fracture is sparse. A 65-year-old male suffered a fall from a height of six meters. Initial X-ray of the chest shows left-sided high-riding diaphragm and CT scan proves anterior cartilage fracture, posterolateral serial rib fractures, traumatic intercostal pulmonary hernia, avulsion of the diaphragm, and 7th thoracic vertebral fracture. An exploratory thoracotomy was performed and the rupture of the diaphragm, creating a two-cavity injury, had been re-fixed, the pulmonary hernia was closed, and locking plate osteosyntheses of the fractured ribs including the costal arch were performed. We generally recommend surgical therapy of the thorax to restore stability in this severe injury entity. The spine was fixed dorsally using a screw-rod system. In conclusion, this thoracovertebral injury entity is associated with high overall injury severity and life-threatening thoracoabdominal injuries. Since two-cavity traumata and particularly diaphragmatic injuries are often diagnosed delayed, injuries to the costal arch can act as an indicator of severe trauma. They should be detected through clinical examination and assessment of the trauma CT in the soft tissue window.

##  

**Figure 1:**
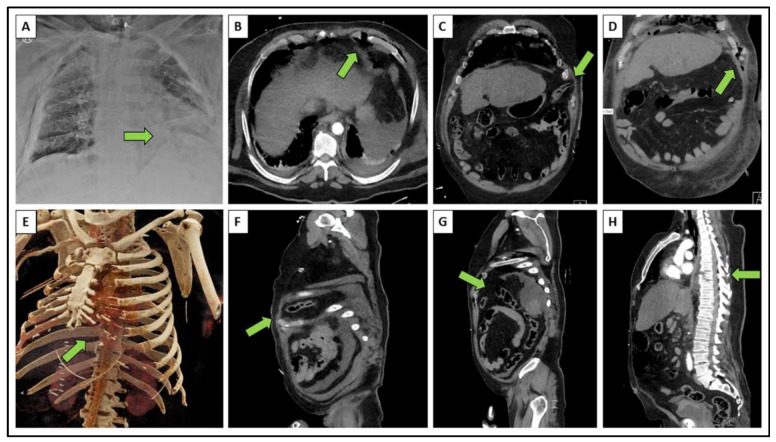
Initial X-ray and whole body CT scan: (**A**) anterior-posterior view, (**B**) axial CT slice, (**C**,**D**) coronar CT slices, (**E**) 3D-CT reconstruction, (**F**–**H**) sagittal CT slices. The respective injuries are marked with arrows. A 65-year-old male suffered from a fall from a height of six meters. Initial X-ray of the chest shows left-sided high-riding diaphragm (Figure 1A). CT scan proves anterior cartilage fracture (Figure 1B), traumatic intercostal hernia within the widened 7th intercostal space (Figure 1C–F), disruption of the diaphragm creating a two-cavity injury (Figure 1G), and distraction fracture of the 7th thoracic vertebra (Figure 1H). The diagnostics showed additional ipsilateral serial rib fractures posterolateral with involvement of the ribs VI–X. This results in a combined thoracovertebral injury entity, consisting of a disruption of the thoracic spine and the costal arch monolaterally torn in the anterior area with anterior fractures of the ribs VII and VIII at their cartilage conjunction, in addition to their posterolateral fractures. The sternum, the mediastinal organs, the center of the diaphragm, and the great vessels did not show any injuries in this case.

**Figure 2:**
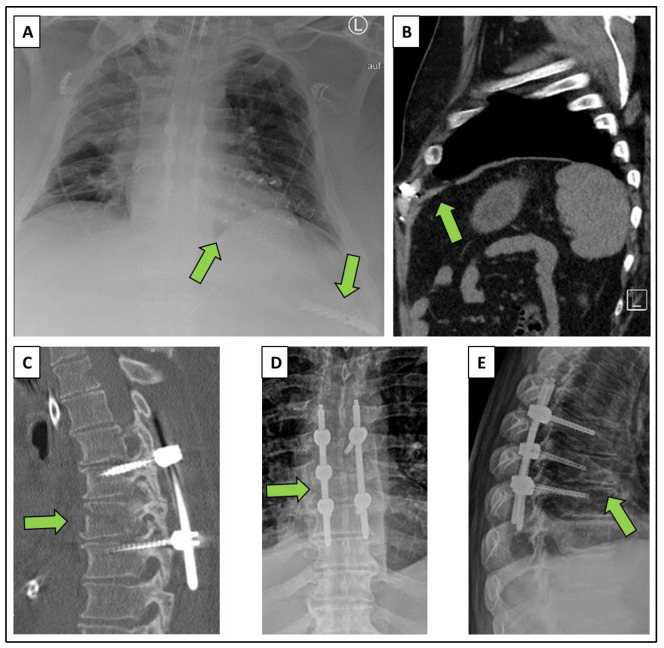
Postoperative X-ray and CT scan: (**A**) thoracic anterior-posterior view, (**B**,**C**) sagittal CT slices, (**D**) anterior-posterior view of the spine, (**E**) lateral view of the spine. The respective injuries and their treatment are marked with arrows. A multistage procedure with a focus on damage control surgery was performed first. The severe rupture of the diaphragm had been fixed in a supine position (Figure 2B). Once the patient had been stabilized via intensive care treatment, the next step on the following day was the fixation of the thoracic vertebral spine in a prone position by dorsal instrumentation with an internal screw rod system (Viper, DePuySynthes, Zuchwil, Switzerland) resulting in anatomic spinal alignment (Figure 2C). Operative treatment had been completed by second look thoracotomy after three days in a lateral decubitus position. Within this procedure, surgical stabilization of rib fractures (SSRF; plates and locked screws) had been included as well as the closure of the intercostal hernia by direct suture of the intercostal muscle layers (Figure 2A). The lower costal margin has been fixed with a locking plate at the confluence of the 7th and the 8th rib. Postoperative X-ray of the chest (Figure 2A) and postoperative CT scan (Figure 2B) show normal intercostal spaces and normal shape of the diaphragm. The patient was able to receive follow-up rehabilitation after discharge and was back to work without any restriction in neurological outcome (5/5 points on Glasgow Outcome Scale). The combined thoracovertebral injury entity seems to be rare and has hardly been researched yet. Injuries with the so-called hyperextension-distraction mechanism of the human trunk are considered to be particularly risky. Individual cases with a ruptured spinal fracture and an anterior rupture of the chest wall with torn sternum are rarely described, probably because most patients already die at the accident scene [1]. A similar accident mechanism can analogously lead to a rupture of the thoracic wall and a simultaneous spinal column fracture [1,2,3]. This case indicates that hyperextension fractures of the thoracic spine may result in high injury severity and overall life-threatening thoracoabdominal injuries once the lower ribs at the costal margin show a fracture at the same time. The energy apparently continued through the thoracoabdominal organs over the intercostal space to the costal arch. This created an unstable situation for the anterior and lateral chest wall. We see the restoration of the stability as a supporting argument for our surgical treatment strategy of the thorax in case of this severe combined injury entity. Equivalently, operative stabilization was recommended for similar injury patterns [4,5,6]. The peculiarity of the injury combination of a distraction injury to the thoracic spine plus a fracture of the costal arch is above all the risk of two major accompanying injuries: an intercostal tear with consecutive lung herniation and a diaphragmatic tear leading to a consecutive two-cavity injury. The indicator function for a severe trauma is again underlined: despite the severity of the associated injuries, two-cavity traumata and particularly diaphragmatic injuries are often diagnosed only secondarily and delayed [4,5]. Because of their association with high overall injury severity, injuries to the costal arch should be detected or excluded by thorough physical examination and assessment of the trauma CT in the soft tissue window. This is rather unique in chest wall trauma and makes a difference to serial rib fractures in their shaft segment. Since we assume an underestimation of this combined injury entity, this clinical recommendation could lead to improved detection of potential associated life-threatening accompanying thoracoabdominal injuries.

## Data Availability

All data generated or analyzed during this study are included in this published article.
